# Exploring anthropometric, biochemistry and nutritional attributes in overweight and obese women: Insights from the Serbian cohort: A pilot study

**DOI:** 10.5937/jomb0-52980

**Published:** 2025-06-13

**Authors:** Anka Petrović, Nevena Ivanović, Neda Milinković, Danijela Milenković, Snežana Polovina, Milica Zeković, Brižita Đorđević

**Affiliations:** 1 University of Belgrade, Faculty of Pharmacy, Department of Bromatology, Belgrade; 2 University of Belgrade, Faculty of Pharmacy, Department of Medical Biochemistry, Belgrade; 3 University of Belgrade, Faculty of Pharmacy, Department of Physics and Mathematics, Belgrade; 4 Clinical Center of Serbia, Clinic for Endocrinology, Diabetes and Diseases of Metabolism, Belgrade, Serbia; 5 University Business Academy, Faculty of Pharmacy Novi Sad; 6 University of Belgrade, National Institute of Republic of Serbia, Institute for Medical Research, Center of Research Excellence in Nutrition and Metabolism, Belgrade

**Keywords:** anthropometric measurements, BMI, Castelli's risk ratio, obesity, VAI, antropometrijska merenja, BMI, Kastelijev indeks rizika, gojaznost, VAI

## Abstract

**Background:**

Anthropometric measurements (AMs), such as waist circumference and body mass index (BMI), are dominant indicators of overweight and obesity. Nevertheless, other AMs, such as waist-hip ratio, waist circumference and indices of visceral adiposity index (VAI), body mass fat index (BMFI), cardiometabolic index (CMI) and waist-to-height ratio (WtHR) are more significant and discriminatory than BMI in predicting cardiometabolic risk.

**Methods:**

This pilot study aimed to comprehensively investigate the anthropometric, biochemical and nutritional characteristics of a sample of overweight and obese women in Serbia, focusing on potential differences related to AM and its association with specific biochemical markers.

**Results:**

The final analytical sample consisted of 57 females (average age 37.16±7.27 years, range: 21-55 years). A strong positive correlation was observed between triglycerides (TG) and both the VAI (r=0.896, p<0.001) and the CMI (r=0.896, p<0.001), both with high statistical significance. Conversely, a strong negative correlation was found between high-density lipoprotein cholesterol (HDLc) and both VAI (r=-0.601, p<0.001) and CMI (r=-0.566, p<0.001). Systolic blood pressure (SBP) and diastolic blood pressure (DBP) were significantly positively correlated with both WtHR and BMFI: SBP and WtHR (r=0.343, p=0.009), SBP and BMFI (r=0.310, p=0.019), DBP and WtHR (r=0.368, p=0.005), and DBP and BMFI (r=0.377, p=0.004). The analysis of biochemical parameters indicated no significant differences between groups, except for TG levels, which were significantly higher in the obese group (p=0.045).

**Conclusions:**

Although the AMs of the subjects were significantly different, energy intake and macronutrient intake were not significantly different. We did not find significant differences in the intake of most vitamins or minerals between the groups. Additionally, there is inadequate intake of essential nutrients such as vitamin D, iron and selenium, which are critical for overall health. Both groups did not meet the recommended folate intake, which may increase the risk of fetal neural tube defects.

## Abstract (sr)

**Uvod:**

Antropometrijska merenja (AM), kao što su obim struka i indeks telesne mase (BMI), predstavljaju dominantne pokazatelje prekomerne težine i gojaznosti. Ipak, druga AM, kao što su odnos struka i kukova, obim struka i indeksi visceralnog indeksa adipoznosti (VAI), indeks telesne masti (BMFI), kardiometabolički indeks (CMI) i odnos struka prema visini (WtHR), pokazuju veći značaj i diskriminativnu vrednost od BMI-a u predikciji kardiometaboličkog rizika.

**Metode:**

Ova pilot studija je imala za cilj sveobuhvatno istraživanje antropometrijskih, biohemijskih i nutricionih karakteristika na uzorku žena sa prekomernom težinom i gojaznošću u Srbiji, sa fokusom na potencijalne razlike pove zane sa antropometrijskim merenjima i njihovom povezanošću sa specifičnim biohemijskim markerima.

**Rezultati:**

Konačan analitički uzorak je činilo 57 žena (prosečna starost 37,16±7,27 godina, raspon: 21-55 godina). Zabeležena je snažna pozitivna korelacija između triglicerida (TG) i VAI (r=0,896, p<0,001) i CMI (r=0,896, p<0,001), obe sa visokom statističkom značajnošću. Nasuprot tome, pronađena je snažna negativna korelacija između holesterola lipoproteina visoke gustine (HDLc) i VAI (r=-0,601, p<0,001) i CMI (r=-0,566, p<0,001). Sistolni krvni pritisak (SBP) i dijastolni krvni pritisak (DBP) su bili značajno pozitivno korelisani sa WtHR i BMFI: SBP i WtHR (r=0,343, p=0,009), SBP i BMFI (r=0,310, p=0,019), DBP i WtHR (r=0,368, p=0,005) i DBP i BMFI (r=0,377, p=0,004). Analiza biohemijskih parametara nije pokazala značajne razlike između grupa, osim u nivou TG, koji je bio značajno viši u grupi gojaznih (p=0,045).

**Zaključak:**

Iako su AM vrednosti ispitanica bile značajno različite, unos energije i makronutrijenata nije se značajno razlikovao. Nismo pronašli značajne razlike u unosu većine vitamina ili minerala između grupa. Pored toga, zabeležen je nedovoljan unos esencijalnih nutrijenata kao što su vitamin D, gvožđe i selen, koji su ključni za opšte zdravlje. Obe grupe nisu dostigle preporučeni unos folata, što može povećati rizik od defekata neuralne cevi kod fetusa.

## Introduction

In recent years, the obesity epidemic has become a widely recognised public health problem [1]. The prevalence of obesity and overweight has increased worldwide and has doubled in recent decades. The prevalence of obesity in Serbia (23.6%) is almost twice as high as the global prevalence of obesity (12%), but the prevalence of overweight (36.9%) is almost identical (39%). According to a report by the Serbian Institute of Public Health, overweight and obesity are major risk factors for the development of chronic noncommunicable diseases such as type 2 diabetes and cardiovascular disease, while approximately 40% of all deaths in Serbia are due to cardiovascular disease [2]. Excessive food intake, often combined with a lack of physical activity, which leads to an imbalance between caloric intake and energy expenditure, is considered one of the main causes of obesity and, thus, noncommunicable diseases.

Anthropometric measurements, including waist circumference (WC) and body mass index (BMI), are important indicators of obesity and overweight. These measurements provide a quantifiable measure of excess body fat and help assess the risk of obesityrelated comorbidities or cardiometabolic risks [3]. Obesity is usually diagnosed by measuring BMI. Thus, overweight is defined as a BMI of 25–29.9 kg/m^2^, and obesity is defined as a BMI of more than 30 kg/m^2^ [4]. However, BMI does not distinguish between fat mass, fat-free mass and fat distribution, which may lead to underestimating body fat.

Nevertheless, other anthropometric measurements, such as WC, waist to hip ratio (WHR), body fat mass (BFM) and body fat distribution (BFD), are more significant and discriminatory than BMI in predicting cardiometabolic risk [5]. Abdominal adiposity as a predictor of central obesity is defined by the measurement of WC (with a cut-off point of 88 cm in women and 102 cm in men), which correlates better with visceral adipose tissue than BMI [6] [7] [8] [9]. According to the working group of the International Atherosclerosis Society and the International Chair of Cardiometabolic Risk, reducing WC in the adult population is not only more important than lowering body weight or BMI but also the most important treatment goal to mitigate adverse health outcomes [7]. However, data from the literature have shown that anthropometric characteristics can vary widely in different populations and are influenced by genetic and environmental factors [10]. Furthermore, adverse metabolic changes and/or organ dysfunction characterised by insulin resistance, dyslipidemia and chronic low-grade inflammation can be observed in overweight and obese individuals [11]. Therefore, routinely available biochemical analyses, such as lipid status and glucose homeostasis parameters, as well as adipocytokine levels, are used to predict cardiometabolic risk and diagnose and monitor obesity treatment [11]. In addition, these metabolic and inflammatory markers correlate more strongly with body fat measurements than with BMI [12].

Diet plays a central role in the development and treatment of overweight and obesity. Eating habits characterised by a high intake of energy-dense, nutrient-poor, high-sugar and high-fat foods and a low intake of fruits, vegetables and fibre contribute significantly to weight gain and obesity [13]. Various socioeconomic, cultural and psychological factors influence the dietary habits of overweight and obese people. Understanding these dietary characteristics is crucial for developing targeted nutritional interventions to promote healthy eating behaviour and weight control [14].

This study aimed to comprehensively investigate the anthropometric, biochemical and nutritional characteristics of overweight and obese women in Serbia. The second aim was to distinguish differences between overweight and obese women in terms of anthropometric measures and their relationships with biochemical markers.

## Materials and methods

### Study design

Data for this pilot study were collected from June 2018 to November 2018. A total of fifty-seven healthy female volunteers of reproductive age between the ages of 21 and 55 were recruited for this study through a public announcement at the Department of Bromatology at the Faculty ofPharmacy in Belgrade. All participants were provided with recruitment materials that included detailed information regarding the study’s aim, all procedures involved, expectations from them, and information about their rights. The recruitment process consisted of four stages: initially screening potential candidates according to predetermined criteria and inviting female individuals to participate in the ongoing research project. These participants were then given comprehensive details about the study’s objectives, responsible parties, the activities involved, and the security measures to safeguard personal information. Finally, ethical and scientifically sound consent was requested. The inclusion criteria were female sex, age between 21 and 55 years, BMI greater than 25 kg/m^2^, and willingness to participate in this study. The exclusion criteria for our study were pregnancy and lactation, any chronic disease, viral or bacterial infections, and the use of any medications or dietary supplements in the three months before and during the study. As gleaned from both participant-provided data and available health records, the study’s collective estimated Charlson Comorbidity Index (CCI) had a median of zero, indicating a minimal comorbidity burden, with the highest score being one, indicating a notably inconspicuous presence of significant health issues. Personal information, sociodemographic details, preexisting medical conditions, daily physical activities, and data regarding dietary habits were collected via structured questionnaires through faceto- face meetings at the Department of Bromatology at the Faculty of Pharmacy in Belgrade.

### Anthropometry and body composition analysis

Blood pressure readings and anthropometric measurements were taken after the subjects were introduced to the study protocol. Systolic and diastolic blood pressure (SBP and DBP) values were measured via an automated oscillometric measurement device, Omron, model HEM-7120-AF (Omron, Co., Ltd., Tokyo, Japan). The results were recorded as the arithmetic mean of three repeated measurements. Before these measurements, participants were seated and allowed to rest quietly for at least five minutes before the initial BP reading.

All measurements were taken from the right arm, and consistent-sized adult cuff values were used. The same researcher measured each participant’s blood pressure, with 5 to 10-minute intervals between measurements [15].

All participants’ anthropometric parameters (body weight, height; waist, chest and hip circumference measurements) were taken at the Department of Bromatology at the Faculty of Pharmacy in Belgrade. Anthropometric measurements were taken while the participants stood in their light underwear with their shoes removed. A SECA 213 stadiometer (SECA, Hamburg, Germany) and an InBody 270 body composition analyser (InBody Co., Seoul, Korea) were utilised to measure body height (in cm) with a precision of 0.1 cm and body weight (in kg) with an accuracy of 0.1 kg, respectively. Moreover, a SECA 201 tape measure (SECA, Hamburg, Germany) was used to obtain waist and hip circumference measurements (in cm) following World Health Organization (WHO) guidelines [16]. Body mass index (BMI) was calculated as weight (kg)/(height (m))^2^, and participants were classified according to WHO criteria [17].

Body composition assessment (percentage of body fat: PBF; body fat mass: BFM; skeletal muscle mass: SMM) was performed via the bioelectrical impedance analysis (BIA) method via an InBody 270 body composition analyser (InBody Co., Seoul, Korea). The measurements were conducted following the manufacturer’s guidelines. The participants were instructed to stand upright on the four electrodes for the feet and place their hands on the four electrodes on the handles, avoiding contact between the torso and arms and between the legs [18].

### Dietary intake assessment

Dietary intake was calculated based on 3-day food intake records. This process was conducted through in-person interviews in accordance with a standardised protocol. The recall interviews were scheduled on non-consecutive days, with two recalls occurring on weekdays and one on a weekend day to ensure a representative dietary assessment and account for intrasubject dietary variability. Information regarding the types of foods or dishes and details such as the time and cooking/processing methods, quantities consumed, and place of consumption were systematically recorded in a designated survey form in chronological order. The participants were instructed to quantify portion sizes based on natural units, packaging information for commercial products and household measures.

Furthermore, to increase the accuracy of portion size estimation, interviews were conducted in conjunction with the Food Atlas, a visual reference tool containing colour photographs depicting various portions of commonly consumed foods and dishes. These photographs were selected based on prior national studies and included four to nine serving sizes for each item, measured via calibrated digital scales. The dietary recall process was facilitated by trained professionals following a standardised protocol. Each interview typically lasted for 15–30 minutes. The questionnaires were processed and analysed via the innovative software-based Diet Assess & Plan platform as part of the dietary data collection and comprehensive nutritional assessment [19]. In this process, the National Serbian Food Composition Database, which adheres to the standards of the European Food Composition Resource (EuroFIR AISBL), was employed for the conversion of food consumption data into estimations of energy, macronutrients, and micronutrients [20].

To evaluate micronutrient adequacy, the dietary recall data provided by the participants were subjected to a comparative analysis against age- and sex-specific nutritional recommendations. These recommendations were based on Dietary Reference Values (DRVs) as proposed by the European Food Safety Agency (EFSA), which serve as reference standards. This approach ensured a robust assessment of micronutrient intake concerning established dietary guidelines and standards.

### Biochemical parameters

Blood samples were collected via venipuncture in the morning (between 7:00 a.m. and 9:00 a.m.) by trained medical staff. Blood was drawn after the participants fasted for 12 hours overnight and fasted without prior physical activity or smoking. A BD Vacutainer (Beckton Dickinson, U.K. Ltd., Oxford, UK) and standard wire gauge 22 (SWG) were used in a closed system for venipuncture. A BD vacutainer system with ethylenediaminetetraacetic acid (EDTA) was used for plasma separation (2.5 mL), and a vacutainer with a serum separator gel (BD Vacutainer® SST™ Tubes, 5 mL) was used to obtain the serum. The plasma samples were stored at + 4°C and then centrifuged at 1500 × g for ten minutes. For serum separation, blood samples were centrifuged for 10 minutes at 3000 rpm at room temperature immediately after the coagulation period (approximately 45 minutes).

Serum samples were analysed on an Olympus AU400 biochemistry analyser (Beckman Coulter Biomedical GmbH, Hamburg, Germany) via standardised spectrophotometric methods and commercial reagents (Beckman Coulter Biomedical GmbH, Hamburg, Germany, and BioSystem, Barcelona, Spain) for the following parameters: high-density lipoprotein (HDL cholesterol) (enzyme test/cholesterol esterase, cholesterol oxidase, anti-lipoprotein (HDL) antibody) and triglycerides (enzyme test/lipase, glycerol phosphatase).

25-Hydroxyvitamin D concentrations were determined via a chemiluminescence immunoassay (CLIA) on an Access 2 immunochemistry analyser (Beckman Coulter Biomedical GmbH, Hamburg, Germany).

The following parameters were determined via RnD Systems ELISA tests (RnD Systems, Inc., USA): human TNF-alpha; human IL-6; human IL-17; human IL-2; human leptin; and human adiponectin/Acrp30.

A semiautomatic Ryto RT 2600C Microplate Washer device was used to wash the plates for ELISA, and the obtained absorbances were read on a semi-automatic Rayto RT-6100 microtiter plate reader.

All samples were analysed at the Laboratory of Medical Biochemistry Analysis, Faculty of Pharmacy, University of Belgrade, Serbia.

### Indices for the prediction of cardiometabolic risk factors

Several indices have been created to reflect cardiometabolic risk: the waist-to-hip ratio (WHR), the waist-to-height ratio (WtHR), and the body mass fat index (BMFI), which adjusts BMI based on body composition and WC. Furthermore, additional predictive indices for MetS that involve both anthropometric and biochemical measurements have been developed, such as the visceral adiposity index (VAI), which combines WC, BMI, triglycerides, and high-density lipo-protein (HDL), and the cardiometabolic index (CMI), which combines the waist-to-height ratio (WtHR) with the TG/HDL-C ratio [21] [22]. The following indices were determined via these formulas [22]:

WHR=\frac{WC(cm)}{HC(cm)}

WtHR=\frac{WC(cm)}{height(cm)}

BMFI=BMI(kg \ m^{-2})\times PBF(\%)\times WC(m)

CMI=WtHR\times \frac{TG(mmol \ L^{-1})}{HDLc(mmol \ L^{-1})}

The VAI was determined using the following formula which is in use in case of female subjects [21]:

VAI=\frac{WC(cm)}{39.58+1.89\times BMI(kg \ m^{-2})}\times

\frac{TG(mmol \ L^{-1})}{0.81}\times \frac{1.52}{HDLc(mmol \ L^{-1})}

Castelli’s risk ratio (CRI) is based on three important lipid profile parameters: triglyceride (TG), LDLc, and HDLc. The CRI-I is calculated as the total cholesterol/HDLc ratio, and the CRI-II is calculated as the LDLc/HDLc ratio [23].

### Ethical approval

The study was approved by the Ethics Commission of the Faculty of Pharmacy, University of Belgrade, Serbia (approval number: 1865/2, Dec 15 2020), and all procedures involving human subjects followed the Declaration of Helsinki guidelines. Informed consent was acquired from each participant in writing.

### Statistical analysis

Statistical analysis was performed via SPSS version 20.0 (SPSS Inc., Chicago, USA). The distribution of continuous variables was examined via the Shapiro-Wilk normality test. The values of descriptive statistics that are normally distributed are presented as the means ± standard deviations (SDs), whereas variables that are not normally distributed are presented as median values [25–75th percentiles]. Depending on the data distribution, the group mean values were compared via the Student’s t-test or the Mann-Whitney U test. Spearman’s rank correlation coefficient is used to measure the correlation between different variables to determine the presence of a monotonic relationship. For all tests, *p*-values of less than 0.05 were considered statistically significant.

## Results

### Characteristics of the study subjects

A total of 65 healthy females were initially approached and assessed against specific eligibility criteria. Five participants reported implausible energy intakes outside the 5000 kcal/day range. They were excluded because they indicated noncompliance with the research protocol and incomplete data submission before the study concluded. Three participants withdrew due to personal issues. As a result, the final analytical sample consisted of 57 females (average age 37.16±7.27 years, range: 21–55 years), reflecting an overall response rate of 87.69%. All of the respondents in this study lived in the city. There were 53 highly educated respondents, four of whom had completed high school. Based on self-reports, 40 individuals were non-smokers, while 15 smoked up to 20 cigarettes in one day, and 2 smoked more than 20 cigarettes a day. Among the 57 participants, 85.96% abstained from all alcoholic beverages, while the rest consumed 1–2 alcoholic drinks per week.

Thirty-eight subjects were overweight (25 BMI< 30), and 19 subjects were obese (BMI 30). The proportion of overweight subjects was 65.52%, and their average age was lower than that of obese subjects (35.82±6.85 vs 39.84±7.54 years, *p*<0.05). The results of appropriate comparison tests demonstrated significant differences in the anthropometric indices between the groups, as shown in Table 1. All values were significantly more significant for the obese group.

**Table 1:** General characteristics and anthropometric indicators of the subjects under study. Notes: BFM, body fat mass; SMM, skeletal muscle mass; BMI, body mass index; PBF, percentage of body fat; WHR, waist-to-hip ratio; WtHR, waist-to-height ratio; BMFI, body mass fat index; VAI, visceral adiposity index; CMI, cardiometabolic index. The values in bold indicate statistical significance.

	Total Sample (*n*=58)	Overweight (*n*=38)	Obesity (*n*=19)	*p*-value
Age (years)	37.16±7.27	35.82±6.85	39.84±7.54	**0.048**
Weight (kg)	79.9 [73.2–89.65]	75.61±6.71	94.2 [87.8–102.5]	**<0.005**
Height (cm)	167.52±6.03	167.93±6.56	166.69±4.86	0.467
BFM (kg)	28.9 [25.3–39.2]	26.52±3.83	42.7 [39.2–51.7]	**<0.005**
SMM (kg)	27.77±2.96	27.02±2.75	29.28±2.83	**0.005**
BMI (kg/m^2^)	28.1 [26.25–32.5]	26.65 [25.4–28.1]	34.6 [32.2–36.5]	**<0.005**
PBF (%)	37.2 [33.8–43.45]	35.28±3.49	45.2 [43.2–51.5]	**<0.005**
Chest circumference (cm)	101 [100–109.5]	100 [97–101.25]	113.84±7.85	**<0.005**
Waist circumference (cm)	87 [81–94]	83.13±5.19	99.32±8.91	**<0.005**
Hip circumference (cm)	111 [107.5–116.5]	109.37±4.83	119 [112–127]	**<0.005**
WHR	0.78±0.08	0.76±0.06	0.82±0.09	**<0.005**
WtHR	0.52 [0.48–0.56]	0.50±0.03	0.60±0.05	**<0.005**
BMFI	8.61 [7.10–13.22]	7.52 [6.81–8.67]	15.03 [13.12–19.42]	**<0.005**
VAI	1.05 [0.64–1.52]	0.94 [0.63–1.25]	1.29 [0.82–1.68]	**0.042**
CMI	0.30 [0.19–0.43]	0.26 [0.17–0.34]	0.40 [0.30–0.55]	**<0.005**

In addition to anthropometric parameters, biochemical indices were also compared, as shown in Table 2. The analysis of biochemical parameters revealed no significant differences, except for the TG values, which were significantly greater in the obese group than in the overweight group. Furthermore, a similar trend was also observed for total cholesterol (TC) values in the overweight group, where the *p*-value was close to the statistical significance threshold. The Castelli’s risk indices were also calculated, summarising the values in Table 2. A significant difference in CRI-I values can be observed when comparing the values obtained for the groups of obese and overweight subjects.

**Table 2:** Biochemical indicators for the subjects under study. Notes: The values in bold indicate statistical significance. HDL, high-density lipoprotein; LDL, low-density lipoprotein; ALT, alanine transaminase; ALP, alkaline phosphatase; AST, aspartate aminotransferase; GGT, gamma-glutamyl transferase; IL, interleukin; CRP, C-reactive protein; RFnm, rheumatoid factor; A/L ratio, adiponectin/leptin ratio; ASLnm, argininosuccinate lyase; CRI, Castelli’s risk indices.

	Total Sample (*n*=57)	Overweight (*n*=38)	Obesity (*n*=19)	*p*-value
Adiponectin (ng/mL)	1.58±0.12	1.60±0.12	1.56±0.11	0.206
Leptin (ng/mL)	5.85±0.46	5.85±0.44	5.74 [5.52–6.14]	0.976
A/L ratio	0.28 [0.26–0.29]	0.27±0.03	0.27±0.03	0.433
IL-17 (pg/mL)	134.15 [98.18–206.46]	147.77 [93.3–305.48]	128.52 [100.19–152.31]	0.416
IL-2 (pg/mL)	196.69 [156.92–309.39]	183.42 [153.69–451.04]	222.54 [162.39–261.39]	0.999
IL-6 (pg/mL)	94.83 [36.61–251.46]	105.6 [35.11–286.27]	72.92 [38.04–192.96]	0.910
TNF-alpha (pg/mL)	104.5 [87.04–152.54]	106.05 [84.18–156.5]	102.95 [88.6–146.2]	0.904
TSH (uIU/mL)<br>0.40–3.50 mIU/L	1.76 [1.13–2.72]	1.43 [0.98–2.79]	1.96 [1.57–2.3]	0.583
Vitamin D (nmol/L)<br>75 nmol/L	92.85±48.06	79.1 [53.09–122.45]	95.98±52.86	0.827
Fasting glucose (mmol/L)<br>4.1–5.9 mmol/L	5.8 [5.4–6.3]	5.6 [5.4–6.13]	6.08±0.81	0.167
Urea (mmol/L)<br>2.8–7.2 mmol/L	4.48±1.16	4.34±1.2	4.77±1.04	0.193
Creatinine (μmol/L)<br>45.0–84.0 μmol/L	68.7±8.03	68.46±8.91	69.18±6.06	0.751
Uric acid (μmol/L)<br>155–357 μmol/L	274.23 [235.04–306.69]	275.33±64.08	306.07±69.52	0.103
ALT <35 U/L	9.7 [6.9–12.2]	9.95 [7.08–11.73]	8.3 [6.1–13.5]	0.520
ALP 30–120 U/L	56.52 [47.26–63.69]	54.72 [46.94–63.2]	58.39 [52.5–64.02]	0.135
AST<35 U/L	17.40 [14.85–21.55]	18.44±4.09	16.7 [14.6–21.5]	0.356
GGT<38 U/L	15.6 [12.25–22.05]	15.0 [12.18 – 19.4]	17.8 [12.2–30.10]	0.310
HDL cholesterol (mmol/L):<br>low <1.03<br>Borderline 1.03–1.55<br>Ideal >1.55	1.26±0.28	1.21 [1.06–1.48]	1.22±0.29	0.654
LDL cholesterol (mmol/L)<br>0.00–3.40 mmol/L	3.73 [3.11–4.7]	3.51 [3.19–4.42]	4.21±1.29	0.559
Serum triglycerides (mmol/L)<br>0.70–1.70 mmol/L	0.69 [0.51–0.91]	0.64 [0.49–0.87]	0.93±0.44	**0.045**
Total cholesterol (mmol/L):<br>Ideal <5.20<br>Borderline high 5.2–6.2<br>High >6.2	4.97±1.04	4.75±0.84	5.39±1.28	0.058
CRP (mg/L) <10 mg/L	2.4 [1.5–4.05]	2.05 [1.3–3.78]	3.2 [1.8–7.1]	0.057
RFnm >20 IU/mL	1.2 [0.75–1.5]	1.2 [0.7–1.55]	1.2 [0.9–1.4]	0.546
ASLnm <200 IU/mL	69.3 [40.85–158.45]	69.25 [37.0–169.88]	73.3 [48.1–147.1]	0.554
Total protein<br>64.0–83.0 g/L	72.0 [68.65–77.0]	71.87±6.81	74.9 [67.1–82.5]	0.233
Albumin 34–54 g/L	48.7 [46.7–51.2]	48.11±3.55	49.58±2.94	0.124
CRI-I	4.05 [3.24–4.65]	3.82±0.94	4.63±1.4	**0.013**
CRI-II	3.17 [2.46–3.92]	2.99 [2.47–3.88]	3.28 [2.37–4.27]	0.416

Table 3 shows the results of food energy intake and carbohydrate, protein, and fat intake. Notably, the mean energy intake value for the obese group was almost 300 kcal greater than that of the overweight group. The mean values for other parameters, such as carbohydrate, protein, and fat intake, were also greater in the obese group. However, it should be noted that none of the values are statistically significant, regardless of the observed differences.

**Table 3:** Energy and nutrient intake.

	Total Sample (*n*=58)	Overweight (*n*=38)	Obesity (*n*=19)	*p*-value
Energy (kcal)	2471.88±659.13	2378.36±620.64	2658.9±710.27	0.131
Carbohydrates (g)	266.98±93.09	254.39±91.04	292.16±94.44	0.150
Protein (g)	92.85±18.6	91.14±19.28	96.28±17.13	0.329
Fat (g)	114.73±35.24	110.69±31.86	122.79±40.93	0.225
Carbohydrates (%)	42.7±7.68	42.21±8.21	43.68±6.58	0.500
Protein (%)	14.53 [13.25–17.85]	14.67 [36.6–47.86]	14.0 [12.9–16.5]	0.476
Fat intake (%)	41.63±6.1	41.87±6.51	41.15±5.31	0.679
Saturated fatty acid (SFA) (g)	39.71±14.72	38.66±14.34	41.80±15.64	0.452
Monounsaturated fat (MUFA) (g)	38.7±12.51	37.26±11.51	41.58±14.2	0.222
Unsaturated fat (USFA) (g)	24.61±9.07	23.13±8.23	27.58±10.13	0.080
Omega-3 polyunsaturated fatty acids<br>(Omega-3 PUFAs) (g)	1.14 [0.73–1.5]	0.99 [0.71–1.42]	1.33±0.53	0.132
Omega-6 fatty acids<br>(Omega-6 PUFAs) (g)	21.15±8.11	19.81±7.35	23.83±9.07	0.077

Regarding mineral intake, the values shown in Table 4, the difference in mineral intake for the groups under observation was not statistically significant.

**Table 4:** Mineral intake. Notes: PRI, population reference intake.<br>(https://multimedia.efsa.europa.eu/drvs/index.htm) (Accessed Dec 30 2022) corresponds to adequate intake (AI), which is used when there is insufficient data to calculate an average intake requirement. The AI is the average nutrient level assumed to be adequate for the population’s needs and is deduced via observations or experiments.

	Total Sample (*n*=58)	Overweight (*n*=38)	Obesity (*n*=19)	PRI (mg/day)	*p*-value
K (mg)	3197.83±669.28	3240.21±681.64	3113.06±653.56	3500 *	0.504
Mg (mg)	334.81±66.29	333.33±61.48	337.75±76.73	300 *	0.815
Na (mg)	3555.97±970.64	3443.29±904.49	3781.35±1081.23	2000 *	0.218
Fe (mg)	11.33 [10.11–12.68]	11.28±2.3	11.38 [10.33–13.43]	16	0.388
Cu (mg)	1.63 [1.4–1.91]	1.64±0.35	1.63 [1.42 – 2.22]	1.3 *	0.467
P (mg)	1429.79±296.95	1387.56±308.64	1514.25±259.32	550 *	0.130
Se (μg)	0.05 [0.04–0.06]	0.05 [0.04–0.06]	0.05 [0.04–0.06]	0.07 *	0.536
Zn (mg)	10.47±2.3	10.13±2.35	11.14±2.11	7.5	0.120

The data concerning vitamin intake through food are provided in Table 5. Except for vitamin B12, where a statistically significant difference with higher values was found for the group of obese subjects, statistically significant differences were not observed for the other vitamins. Nonetheless, it is interesting to note that overweight subjects consumed more vitamin D than obese subjects did; however, this value was still lower than the recommended daily intake. Importantly, for both the obese and overweight groups, the folate intake values were lower than the recommended daily intake values.

**Table 5:** Vitamin intake. Notes: PRI, population reference intake.<br>* (https://multimedia.efsa.europa.eu/drvs/index.htm) (Accessed on Dec 30 2022) corresponds to adequate intake (AI), which is used when there is insufficient data to calculate an average intake requirement. The AI is the average nutrient level assumed to be adequate for the population’s needs, which is deduced via observations or experiments. The values in bold indicate statistical significance. † For calculation, the average energy intake in the MJ was taken.

	Total Sample (*n*=58)	Overweight (*n*=38)	Obesity (*n*=19)	PRI	*p*-value
Thiamine (mg)	1.33<br>[1.09–1.77]	1.43±0.05	1.43±0.46	0.1 mg/MJ<br>(aprox. 1.04 mg)†	0.993
Riboflavin (mg)	1.65±0.47	1.61±0.42	1.73±0.56	1.6	0.390
Niacin (mg)	24.53±6.41	24.28±6.3	26.43<br>[18.61–31.39]	1.6 mg/MJ<br>(aprox. 16.6 mg)†	0.576
Pantothenic acid<br>(mg)	3.05<br>[2.3–4.22]	3.12<br>[2.11–4.22]	3.37±1.17	5*	0.685
Pyridoxine<br>(vitamin B6) (mg)	1.54±0.55	1.59±0.59	1.43±0.46	1.6	0.308
Biotin (mg)	0.02<br>[0.01–0.02]	0.02±0.01	0.02<br>[0.01–0.03]	0.04	0.926
Folate (μg)	261.63±66.16	260.74±61.29	263.41±76.75	330 μg/day	0.887
Cobalamin<br>(Vitamin B12) (μg)	3.49<br>[2.68–4.89]	3.25<br>[2.48–4.03]	4.24<br>[2.88–6.47]	4 μg/day*	**0.034**
Ascorbic acid<br>(Vitamin C) (mg)	125.18<br>[64.39–190.21]	133.75<br>[72.67–193.14]	125.18<br>[57.18–176.95]	95	0.264
Vitamin A (mg)	0.65<br>[0.48–0.79]	0.68±0.21	0.61<br>[0.4–0.71]	0.65	0.148
Vitamin D (μg)	3.23<br>[2.29–5.24]	3.55<br>[2.38–5.35]	2.72<br>[2.19–4.62]	15 μg/day*	0.388
Vitamin E (mg)	19.73<br>[16.75–24.88]	21.54±8.0	19.03<br>[17.21–23.98]	11*	0.986

### Correlations between nutrient Intake, anthropometric parameters, and inflammatory and cardiometabolic indices

Correlations between dietary intake of zinc, selenium, and vitamin C and inflammatory indices, i.e., IL-17, IL-2, IL-6, TNF-alpha, and CRP, are presented in Table 6. No statistically significant correlations, except for a positive correlation between vitamin C and CRP (r=0.345, *p*<0.05), were detected between the intake of these food antioxidants and inflammatory parameters.

**Table 6:** Correlation coefficients between indices of adiposity and metabolic characteristics. Notes: IL, interleukin; TNF, alpha tumour necrosis factor-alpha; CRP, C-reactive protein; r, Spearman’s rank correlation coefficient; *p*, statistical significance of the test. The values in bold indicate statistical significance.

	Zn (mg)				Se (μg)				VITC (mg)			
	Overweight		Obesity		Overweight		Obesity		Overweight		Obesity	
	R	*p*	r	*p*	r	*p*	r	*p*	r	*p*	r	*p*
IL-17 (pg/mL)	0.007	0.968	0.210	0.389	0.203	0.242	-0.171	0.483	-0.029	0.871	0.048	0.844
IL-2 (pg/mL)	-0.031	0.864	0.042	0.864	-0.081	0.654	0.071	0.772	-0.292	0.099	-0.061	0.803
IL-6 (pg/mL)	0.012	0.946	-0.067	0.786	0.028	0.868	-0.191	0.434	0.038	0.821	0.007	0.977
TNF-alpha<br>(pg/mL)	-0.143	0.397	0.242	0.318	0.045	0.793	-0.206	0.398	0.087	0.608	-0.112	0.647
CRP	-0.003	0.984	-0.041	0.871	0.081	0.631	0.035	0.890	0.347*	**0.033**	0.138	0.584

Furthermore, according to the correlation coefficient values between the anthropometric parameters (PBF, BFM, and BMI) and macronutrient intake, there was a positive correlation, but not statistically significant. The highest correlation observed was between carbohydrate percentage intake and fat tissue mass in the group of overweight subjects (Supplementary material, Table 1). Furthermore, these anthropometric parameters and mineral intake correlations were not statistically significant (Supplementary material, Table 2).

The correlations between indices of adiposity and cardiometabolic parameters are presented in Table 7. A strong positive correlation with high statistical significance was observed between TG and VAI (r=0.896, *p*<0.001) and between TG and CMI (r=0.896, *p*<0.001). A strong negative correlation with high statistical significance was also detected between HDLc and the VAI (r=-0.601, *p*<0.001) and between HDLc and CMI (r=-0.566, *p*<0.001).

**Table 7:** Correlation coefficients between dietary antioxidants (Zn, Se, and vitamin C) and inflammatory indices. Notes: SBP, systolic blood pressure; DBP, diastolic blood pressure. The values in bold indicate statistical significance.

	SBP (mm Hg^-1^)	DBP (mm Hg^-1^)	HDLc (mmol L^-1^)	Glycemia (mmol L^-1^)	TG (mmol L^-1^)
WHR	r=0.133 *p*=0.323	r=0.110 *p*=0.413	r=0.045 *p*=0.741	r=0.076 *p*=0.573	r=0.025 *p*=0.852
WtHR	**r=0.343 *p*=0.009**	**r=0.368 *p*=0.005**	r=0.030 *p*=0.825	r=0.167 *p*=0.243	r=0.095 *p*=0.484
BMFI	**r=0.310 *p*=0.019**	**r=0.377 *p*=0.004**	r=0.020 *p*=0.884	r=0.212 *p*=0.113	r=0.170 *p*=0.207
VAI	r=0.186 p=0.166	r=0.096 *p*=0.477	**r=-0.601 *p*< 0.001**	r=0.193 *p*=0.150	**r=0.896 *p*< 0.001**
CMI	r=0.239 *p*=0.073	r=0.153 *p*=0.256	**r=-0.566 *p*< 0.001**	r=0.218 *p*=0.103	**r=0.896 *p*<0.001**

Furthermore, SBP and DBP were significantly positively correlated with WtHR and BMFI: SBP and WtHR (r=0.343, *p*=0.009), SBP and BMFI (r=0.310, *p*=0.019), and DBP and WtHR (r=0.368, *p*=0.005), and DBP and BMFI (r=0.377, *p*=0.004). However, the correlation between adiposity indices and glycemia did not reach statistical significance.

## Discussion

The main objective of this study was to comprehensively assess the dietary intake of macro- and micronutrients in overweight and obese women of childbearing age while exploring the relationships among anthropometric parameters, biochemical parameters and inflammatory factors in these two groups. As expected, all measured anthropometric indices, including PBF, WtHR, BMFI, WHR, CMI, and VAI, were significantly greater in obese individuals than in their overweight counterparts. A recently published study confirmed that subjects of both sexeswith PBF values above the PBF cut-off of more than 37.1% among women had a 2–4-fold higher risk of developing cardiovascular risk factors than did subjects with PBF values below the cut-off [24], suggesting that subjects in the obese group had an increased risk of developing cardiovascular diseases. Recent evidence indicates that anthropometric measurements can help identify women with obesity who are at heightened risk for developing MetS [25]. The diagnosis of MetS combines both anthropometric and biochemical parameters [26]. Given that the amountand distribution of fat mass are closely associated with the development of MetS [27], additional indices based on these factors have been created. These indices include the waist-to-height ratio (WtHR), body mass fat index (BMFI) and waist-to-hip ratio (WHR) [22]. The values that can indicate an increased risk of developing metabolic syndrome are WHR >0.92, WtHR >0.76, BMFI >30.1, VAI >1.94, and CMI >0.84 [22]. The percentages of women who indicated a risk of developing metabolic problems in our sample were as follows: WHR, 7.02%; WtHR and BFMI, 0%; CMI, 3.51%; and VAI, 12.28%. Since the diagnosis of metabolic syndrome incorporates anthropometric and biochemical factors, the VAI is particularly valuable as it integrates measures of body fat distribution (such as WC and BMI) alongside biochemical indicators like triglycerides and HDL cholesterol. In contrast, WtHR is focused exclusively on body composition parameters. Although the CMI was elevated beyond the cut-off value (>0.94) in only a small subset of women, our analysis revealed that both CMI and VAI are more robust predictors of metabolic risk factors compared to other indices, a finding consistent with existing literature [21] [22] [26] [28].

Nevertheless, no significant differences in TC or LDLc levels were found between the two groups. Studies have shown that when conventional lipid parameters (TG, HDLc, LDLc and TC) appear normal, lipid indices such as the CRI-I and CRI-II provide a diagnostic alternative that can predict the risk of cardiovascular events [29] [30]. On the basis of these risk indices, we found a significant difference in atherogenic indices between these two groups. With an estimated value of over 4, the CRI-I value in the obese group was consistent with that reported in other studies [31] [32] and was significantly greater than that reported in the overweight group. Several studies have also reported an association between the CRI-I score and coronary plaque formation [33] [34]. In addition, the CRI-II index was higher in the obese group than the overweight group, although the difference was insignificant. However, it was higher than the upper limit for the normal range reported in the literature, i.e., >3 [32].

Our study examined inflammatory indices, including adipocytokines, in overweight and obese female subjects. Although the adiponectin/leptin (A/L) ratio was lower in both groups, the difference was statistically insignificant. As stated by Frühbeck et al. [35], A/L ratio of 1.0 or higher is considered normal. A ratio below 1.0 but equal to or greater than 0.5 indicates a moderate to medium increased risk, while a ratio less than 0.5 suggests a severe increase in cardiometabolic risk [35]. Based on our findings, which show an A/L ratio of 0.27±0.03 in both groups, we can infer that the participants in our study are at a significantly heightened risk for cardiometabolic complications.

Literature evidence indicates a negative correlation between the A/L ratio and BMI in obese individuals, implying that the A/L ratio tends to decrease as BMI increases. A lower A/L ratio indicates an inflammatory state within adipose tissue and is closely associated with heightened cardiometabolic risk [35].

In addition, the A/L ratio is a more effective parameter for insulin resistance than adiponectin or leptin levels alone in nonhyperglycemic individuals [36] [37]. In contrast, an increase in the A/L ratio was associated with an improvement in insulin sensitivity, suggesting that the A/L ratio may be a useful biomarker for monitoring the effects of lifestyle interventions on metabolic health [35] [37].

The second segment of our research focused on evaluating the participants’ dietary patterns and how they relate to their anthropometric and metabolic profiles. As shown in Table 4, it is evident that the obese group had elevated energy intake alongside higher consumption of carbohydrates, proteins, and fats. These findings are consistent with expectations, as individuals with obesity typically have greater caloric needs, which often result in higher overall food intake [38]. Although the subjects showed statistically significant differences in their anthropometric parameters, energy and macronutrient intake were not significantly different. From the results of the food diary analysis, we can conclude that the percentage intake of carbohydrates was lower in both groups, whereas the total percentage intake of dietary fats was higher than the values recommended by the European Food Safety Authority (EFSA) [39]. According to the results of the present study (Table 4), the average intake of SFAs was greater than the recommended 10% of daily dietary intake [40] [41]. SFAs are associated with increased levels of total cholesterol and low-density lipoproteins in the blood [42]. In addition, studies have shown that consuming more saturated fat than the recommended 10% in the diet is associated with an increased risk of cardiovascular disease and elevated blood triglyceride levels [43]. There is a close relationship between saturated fat intake and the occurrence of inflammation, oxidative stress and lipoperoxidation. These factors contribute to the gradual development of atherosclerosis in blood vessels to varying degrees [44].

Across all participants’ dietary vitamin D intake was consistently below the recommended daily intake of 15 μg [45]. Although the obese group demonstrated a slightly higher vitamin D intake than the overweight group, this difference did not reach statistical significance. This finding aligns with existing literature, which indicates that vitamin D intake is often reduced in individuals with obesity [46] [47] [48]. Moreover, a higher BMI is frequently associated with serum concentrations of 25-hydroxyvitamin D (25(OH)D) [47] [49] [50]. Obesity presents a significant risk factor for vitamin D deficiency and related cardiometabolic risks, primarily due to the fat-soluble nature of vitamin D. As adiposity increases, vitamin D becomes sequestered within the expanded fat mass, thereby diminishing its bioavailability [51] [52]. Despite the suboptimal dietary vitamin D intake observed in both groups, serum vitamin D levels remained within the normal range for all participants. A pilot study of a small cohort of Serbian adults revealed that 60.58% of the subjects were vitamin D deficient (with serum 25(OH)D values less than 50 nmol/L), most of whom were overweight women [53].

In contrast, all the subjects in our study had values of 25(OH)D >75 nmol/L, and the results were not significantly different between the groups studied, with higher values in the overweight group. This outcome may be attributed to the timing of the study, which coincided with periods of increased sunlight exposure and enhanced endogenous vitamin D synthesis (June to November 2018) [54]. Furthermore, studies indicate a significant association between increased gene expression for the vitamin D receptor in obese people; thus, in addition to determining the vitamin D level in obese people, the level of this receptor should also be examined. This is the only way to recognise the true picture of biologically active and functional vitamin D [55] [56]. According to our results, the analysis of biomarkers to assess nutritional status must be combined with information on food consumption, i.e., although vitamin D intake was probably insufficient in the study participants, the immediate health consequences due to sunlightinduced production of the vitamin may not be of concern.

Apart from vitamin B12, where a statistically significant difference was found with higher values in the obese group, no statistically significant differences were observed for the other vitamins. This result can be explained by the fact that obese subjects consumed more vitamin B-rich foods, such as meat, dairy products and eggs. Recent research has shown that the B12 concentration increases as BMI increases [57]. Preclinical and clinical studies have shown that adequate intake of vitamin B12 can lower TG and LDL cholesterol levels [58]. These results suggest that adequate intake of vitamin B12 may play a role in preventing dyslipidemia and cardiovascular disease [58] [59].

In our study, the average folate intake was 261.63±66.16 μg/day, which is slightly higher than the previously reported intake of 211.00±81.06 μg/day among Serbian women of childbearing age [60]. The earlier study primarily involved women who were predominantly of normal weight (70.18%), with smaller proportions classified as overweight (20.08%) or obese (5.57%). Despite this slight increase, inadequate folate intake remains a concern, as it is a well-recognised preventable risk factor for congenital anomalies and other adverse pregnancy outcomes. Extensive research, including observational and interventional studies, has consistently demonstrated the protective effect of periconceptional folic acid supplementation in reducing the risk of both the occurrence and recurrence of neural tube defects [61]. Although the folate intake observed in our study is somewhat higher, it still falls short of the recommended levels, especially when considering the increased energy intake reported by the participants. This suggests that these women may still be at risk for fetal neural tube defects and other neurological disorders, underscoring the need for continued attention to adequate folate intake during the periconceptional period [61].

Regarding mineral intake, as shown in Table 5, we can conclude that the subjects did not consume an adequate amount of selenium-rich foods, as their selenium intake was well below the levels recommended by the European Food Safety Authority (EFSA) (70 μg/day for women) [62]. Selenium is an antioxidant micronutrient in various foods, including fish, cereals, meat and vegetables [63]. Previous studies have shown that the selenium intake of the Serbian population is inadequate at only 40.9 μg/day and that foods of animal origin account for the largest proportion of total selenium intake at 31.7 μg/day [64]. Selenium supplements are used in livestock feed to improve animal health and performance. Adding selenium to animal feed has increased the selenium content of meat, eggs and milk. Although selenium supplementation in livestock feed, which began in Serbia in 2000, has led to an increase in the selenium content of meat, eggs, and milk [65] this has not translated into sufficient selenium intake among our study participants. Wang et al. reported that in the general adult population, obesity and the degree of obesity are associated with low dietary Se intake [66]. Increasing dietary Se intake by 1 μg/kg/day resulted in a 3–6% decrease in body fat percentage [66]. In our study, obese and overweight women had extremely low selenium intakes, emphasising the importance of additional selenium intake or supplementation. However, we did not find a significant correlation between dietary intake of antioxidants (selenium, zinc and vitamin C).

In addition to selenium, iron intake was below the recommended values and the same in both groups (11.33 [10.11–12.68] mg/day). The adequacy of iron intake was determined on the basis of the recommendations of the European Food Safety Authority (EFSA) for the general population (16 mg/day for women) [67]. A recent European study involving women of childbearing age from 12 countries, including Serbia, reported that the median or mean iron intake was between 7.6 and 9.9 mg/day, which is less than the recommended amount [68]. Most studies on the relationship between obesity and iron deficiency have focused on specific hematologic and biochemical markers [69] [70]. Inadequate dietary iron intake can lead to iron deficiency and anaemia, especially in overweight or obese women. These individuals are at risk of reduced iron absorption and utilisation, which can exacerbate the development of anaemia. In addition, research suggests that improving iron absorption with ascorbic acid is less effective in overweight and obese women than in normal-weight women, further highlighting the complex relationships among obesity, iron deficiency and anaemia [71] [70]. Anaemia is also associated with BMI and waist circumference in Chinese women, underscoring the importance of maintaining a healthy weight to prevent this condition [72]. Our participants’ insufficient dietary iron intake may contribute to a heightened risk of anaemia. This finding highlights the critical need for tailored nutritional strategies to mitigate iron deficiency in the vulnerable population of overweight and obese women. Conversely, the obese participants in this study consumed higher sodium and phosphorus levels. Excessive salt intake is a well-established, independent risk factor for elevated blood pressure, which is critical in the onset of cardiovascular diseases. Beyond its impact on blood pressure, numerous studies have demonstrated that consuming more than 3 grams of table salt daily is significantly linked to a heightened risk of cardiovascular, cerebrovascular, and renal disease [73] [74]. In the Republic of Serbia, the prevalence of hypertension is 46.5%, with a systolic blood pressure of 140 mm Hg and a diastolic blood pressure of 90 mm Hg in adults aged 18 years and older. This is mainly due to the high salt intake and its content in processed, ready-to-eat foods [75]. The results of this study suggest that both groups of subjects consumed an excessive amount of salt, i.e., sodium, with the obese group exhibiting even higher levels of intake. This finding underscores the potential role of excessive sodium consumption as a risk factor for the development of cardiovascular disease within the studied population group [76].

Based on the results obtained, we conclude that dietary phosphorus intake (1514.25±259.32 mg/day) is 2.5 times greater than adequate intake [77]. Several recent studies have revealed associations between high phosphorus intake or high serum inorganic phosphate concentrations and morbidity and mortality [78] [79]. High phosphorus intake is associated with a variety of diseases, particularly cardiovascular disease, due to a chronic phosphorus-rich diet. In obesity, elevated inorganic phosphate levels in the bloodstream have been observed, though the precise mechanisms remain elusive. Excessive intake of sugars, sugar-sweetened beverages, and other carbohydrate-rich sources promotes the absorption of glucose and inorganic phosphate, which may subsequently enhance insulin signalling in adipocytes, driving the intracellular accumulation of inorganic phosphate [80].

Several limitations of this study warrant careful consideration. The relatively small sample size challenges the findings’ external validity, potentially limiting their generalizability to a broader population. Additionally, the reliance on self-reported dietary intake data introduces the possibility of recall bias and underreporting, particularly for less prominently consumed items like vegetables and spices. Despite rigorous adherence to the study’s dietary assessment protocols, these factors may have introduced variability in the accuracy of the reported data. Furthermore, the study’s short duration limits the ability to assess the long-term consistency of the observed correlations. Future research should extend the observation period beyond one year to determine whether these patterns remain stable over time.

## Conclusion

This study contributes to the emerging body of research by providing a detailed analysis of dietaryintake, anthropometric parameters, and biochemical indices in overweight and obese Serbian women of childbearing age. Although there were statistically significant differences in anthropometric parameters such as BMI and PBF between the overweight and obese participants, emphasising the increased cardiovascular risk in the obese group, there were no significant differences in the intake of most vitamins and minerals between the groups. However, both groups had inadequate intakes of essential nutrients such as vitamin D, selenium and iron, which are critical for overall health. Both groups did not meet the recommended folate intake, which may increase the risk of fetal neural tube defects. The higher intake of sodium and phosphorus in overweight and obese participants raises concerns about their long-term cardiovascular health, as these elements have been linked to hypertension and other cardiovascular diseases.

Considering that our research is limited by small sample size, future research should consider larger, more diverse populations and longer study periods to validate these results and better understand the longterm effects of dietary intake on health outcomes in overweight and obese women.

In conclusion, this study emphasises the need for targeted nutritional interventions to address the observed deficits and excesses and reduce the risk of cardiovascular diseases and other health complications in overweight and obese women.

## Dodatak

### Funding

The authors received no financial support for this article’s research, authorship, and publication.

### Data availability statement

Data to support the findings of this study is available upon reasonable request from the corresponding author.

### Acknowledgements

Not applicable.

### Conflict of interest statement

All the authors declare that they have no conflict of interest in this work.
